# Nanostructures Stacked on Hafnium Oxide Films Interfacing Graphene and Silicon Oxide Layers as Resistive Switching Media

**DOI:** 10.3390/nano13081323

**Published:** 2023-04-09

**Authors:** Tauno Kahro, Kristina Raudonen, Joonas Merisalu, Aivar Tarre, Peeter Ritslaid, Aarne Kasikov, Taivo Jõgiaas, Tanel Käämbre, Markus Otsus, Jekaterina Kozlova, Harry Alles, Aile Tamm, Kaupo Kukli

**Affiliations:** Institute of Physics, University of Tartu, W. Ostwaldi 1, 50411 Tartu, Estonia

**Keywords:** silicon oxide, hafnium oxide, resistive switching, graphene, stacked nanostructures, atomic layer deposition

## Abstract

SiO_2_ films were grown to thicknesses below 15 nm by ozone-assisted atomic layer deposition. The graphene was a chemical vapor deposited on copper foil and transferred wet-chemically to the SiO_2_ films. On the top of the graphene layer, either continuous HfO_2_ or SiO_2_ films were grown by plasma-assisted atomic layer deposition or by electron beam evaporation, respectively. Micro-Raman spectroscopy confirmed the integrity of the graphene after the deposition processes of both the HfO_2_ and SiO_2_. Stacked nanostructures with graphene layers intermediating the SiO_2_ and either the SiO_2_ or HfO_2_ insulator layers were devised as the resistive switching media between the top Ti and bottom TiN electrodes. The behavior of the devices was studied comparatively with and without graphene interlayers. The switching processes were attained in the devices supplied with graphene interlayers, whereas in the media consisting of the SiO_2_-HfO_2_ double layers only, the switching effect was not observed. In addition, the endurance characteristics were improved after the insertion of graphene between the wide band gap dielectric layers. Pre-annealing the Si/TiN/SiO_2_ substrates before transferring the graphene further improved the performance.

## 1. Introduction

Electronic memristors, characterized by low power consumption and a simple structure, are searched upon the research and development of next-generation nonvolatile computer memories, including resistive random access memory (RRAM) prototypes. The artificial mixing of different oxides in order to tailor their useful physical properties could allow the extension of their application areas, including memory materials, wherein graphene, as a component layer, may enhance flexibility and transparency [1]. Memristive devices can be designed based on very different metal oxide host media, including hafnium oxide [1]. Combinations between different oxides, such as SiO_2_, HfO_2_, Ta_2_O_5_, and Al_2_O_3_, with two-dimensional crystalline layers, primarily graphene, appear purposeful [2]. An earlier study [2] has shown that the combination of Al_2_O_3_-based RRAM devices with SiO_2_ layers could result in a device exhibiting an increase in the memory window width by a factor of 1000. On the other hand, due to the randomness of conductive filament distribution and strength, the switching behavior of the SiO_2_-based device has remained unstable without additional modifications [2]. It has also been found that graphene, if inserted into the switching media, can reduce power consumption and prevent the growth of uncontrollable conductive filaments [3,4], although graphene alone could not effectively improve the uniformity of the switching characteristics [2]. Alternative two- or one-dimensional nanomaterials, such as MoS_2_ layers or metallic quantum dots [2], respectively, have been examined in order to improve the uniformity, switching stability, and endurance of resistively switching cells. In another study, graphene oxide layers, covered by a sputtered Zr-doped SiO_2_ film, were examined as a switching medium, demonstrating superior performance compared to that of analogous structures without graphene oxide [5]. By incorporating graphene oxide and thin HfO_x_ films in flexible PET/ITO/HfO_x_/GO/Al ReRAM devices, stable and uniform bipolar resisting switching behavior was achieved [6]. It was also shown that the use of graphene as an interfacial layer avoids the structural irreversible damage of the HfO_2_ layer, allowing unipolar switching in Si/HfO_2_/Gr/Ti/Au devices not detected in the same devices without graphene [7]. Using graphene as an electrode material, the ON/OFF ratio was improved by one order of magnitude without increasing the switching power, as demonstrated in Si/SiO_2_/Ti/Pt/ta-C/Gr/Cr/Au devices [8].

Chemical vapor deposition (CVD) is a widely used method for the synthesis of large-area graphene on metal substrates, mainly on Cu foils. The CVD-grown graphene has often been formed as polycrystalline layers, possessing electronic mobility values lower than those expected from single-layer graphene [9,10]. Recently, some achievements have been made in this field. Namely, single-crystalline, large-area, and fold-free graphene was chemical vapor deposited from an ethylene precursor, instead of methane, on a Cu-Ni(111) alloy instead of Cu foils [11]. To fabricate graphene-based electronics, graphene must be transferred from metal foils onto functional substrates. Specifically, wet-chemical [12], metal-assisted [13], or electrochemical (H_2_ bubbling) [14] exfoliation and transfer methods are mostly used.

The atomic layer deposition (ALD) of oxides, allowing accurate control over the layer thickness, could possibly be regarded as the most promising method for the synthesis of insulating films, constituting metal–insulator–metal, i.e., MIM-like structures, including those containing graphene. However, growing uniform dielectric layers directly on top of graphene is still a challenging task, primarily due to the chemical inertness of single-layer graphene [15,16]. Plausibly, film growth techniques based on physical vapor deposition, such as electron beam evaporation, which is less dependent on the chemical bonding between the oxide and graphene, may allow one to feasibly build stacks of continuous oxide films containing graphene [17].

In addition to the fact that dielectric films may not nucleate and grow uniformly on the chemically inert carbon surface, the chosen growth conditions can cause complete destruction of the graphene layer [18,19,20,21]. For instance, care should be taken when using ozone or oxygen plasma as oxidizing precursors [22,23]. It has been shown that in the ozone-assisted ALD process of Al_2_O_3_, carried out at 200 °C on graphene, a strong defect-induced D-band appeared in the Raman spectra due to functional groups (such as epoxides) formed on the carbon sites. The latter observation differs from the reports, according to which the graphene might remain resistant to the ozone in the ALD process, although it was carried out at considerably lower temperatures (80 °C) [22]. The use of oxygen plasma in the ALD of metal oxides onto graphene can also damage the graphene and degrade its electrical properties. To avoid this, a sacrificial graphene layer can be used, as shown before [23].

The nucleation and growth of metal oxide layers on graphene sheets can be proliferated by lowering the deposition temperature and, thereby, increasing the density of the adsorption sites, which is also the case with the chemical vapor phase deposition method. Low-temperature fabrication routes are recommended due to the enhanced flexibility in designing semiconductor structures. For instance, stacked HfO_2_/SiO_2_ gate oxide films have been successfully fabricated, even at room temperature, by neutral beam-enhanced atomic layer deposition [24]. Notably, we have earlier grown HfO_2_ films successfully in a plasma-assisted ALD process from a hafnium alkylamide precursor and plasma-generated from O_2_, after the pre-treatment of graphene with low-temperature cycles of water-assisted ALD of HfO_2_ [4]. The process developed in the latter study was integrated into the present experimental work in order to fabricate graphene-based functional material stacks.

The present study is devoted to the investigation of the integrity and functionality of the graphene layers transferred to ALD-grown SiO_2_-insulating dielectric films in both as-deposited and annealed states. Spectroscopic and microscopic studies were conducted before and after the growth of dielectric HfO_2_ and SiO_2_ layers on graphene to evaluate the structural quality of the transferred graphene. In order to examine the potential usefulness of the graphene transferred to chemically deposited silicon oxide surfaces embedded between silicon/metal oxide layers, resistively switching structures were devised with the aim of evaluating their primary current–voltage and endurance characteristics.

## 2. Materials and Methods

### 2.1. Bottom SiO_2_ Dielectrics on Top of Si/TiN Substrates

The conductive Si(100) wafers were boron-doped to concentrations of 5 × 10^18^–1 × 10^19^ cm^−3^ and coated with a ~10 nm crystalline TiN layer by pulsed chemical vapor deposition using a batch TiCl_4_/NH_3_ process [25,26] at temperatures of 450–500 °C in an ASM A412 Large Batch 300 mm reactor at Fraunhofer IPMS-CNT.

For the deposition of amorphous SiO_2_ dielectric films on top of Si/TiN substrates, hexakis(ethylamino)disilane (99.995%-Si, Strem Chemicals inc., Newburyport, MA, USA), i.e., Si_2_(HNC_2_H_5_)_6_, and ozone were used as precursors [27]. The dielectric silicon oxide films were grown in an in-house-built hot-wall flow-type ALD reactor [28] at 240 °C. The cycle times used were 2/2/5/5 s, denoting the sequence of the Si precursor, pulse–purge–ozone pulse–purge. The films were grown by applying 75 cycles.

### 2.2. CVD Graphene and Transfer

Graphene was grown on 25 µm-thick polycrystalline copper foil (99.5%, Alfa Aesar, Ward Hill, MA, USA) in an in-house-built CVD reactor. The foil was, prior to the growth, annealed at 1000 °C in an Ar and H_2_ (both 99.999%, Linde Gas, Tallinn, Estonia) flow for 60 min, then exposed to a mixture of 10% CH_4_ (99.999%, Linde Gas) in Ar at 1000 °C for 120 min, and cooled down to room temperature in the Ar flow. The as-deposited graphene was transferred from the copper foil to Si/TiN wafers, pre-covered by ALD-grown SiO_2_, using poly(methyl-methacrylate) (PMMA, molecular weight of ~996,000 g/mol, Sigma Aldrich, Darmstadt, Germany) as a supporting layer in a wet chemical process. The PMMA solution, in chlorobenzene (Sigma-Aldrich), was spin-coated onto one side of graphene/copper/graphene to a thickness of around 120–150 nm, and the graphene on the uncoated side was removed by Ar^+^ plasma. Thereafter, the copper foil was dissolved in a 1M ammonium persulfate (Sigma Aldrich) solution overnight. To remove the residual etchant, the floating graphene/PMMA film was soaked several times in deionized water baths and transferred to dielectric substrates. The samples were dried in air overnight and then heated at 100 °C to improve the contact between the graphene and the substrate. The PMMA layer was dissolved in dichloromethane (Sigma-Aldrich), and the graphene samples were carefully rinsed with 2-propanol (Sigma-Aldrich) to remove possible residues.

### 2.3. Upper HfO_2_ or SiO_2_ Dielectrics

To protect graphene against oxygen plasma and promote the growth of the upper HfO_2_ dielectric, a buffering nucleation layer on the surface of graphene was formed in a PicosunTM R-200 Advanced ALD system, at first by soaking in 0.1 s long H_2_O pulses repeated 120 times, whereby the pulses were separated by 0.9 s-long purge periods. The soaking was followed by exposure to 0.1 s long hafnium tetrakisethylmethylamide, Hf[N(C_2_H_5_)(CH_3_)]_4_, i.e., (TEMAHf) (99.99 %, Sigma Aldrich) pulses, which was also repeated 120 times. All TEMAHf pulses were separated by 0.9 s long purge periods. The exposure to the sequence of TEMAHf pulses was, again, followed by 0.1 s long H_2_O pulses repeated 120 times, whereby the pulses were separated by 0.9 s long purge periods. The buffer layer was, thus, formed similarly to the cycling sequence exploited in our earlier study [4]. For the deposition of upper HfO_2_ dielectric layers, 80 ALD cycles were applied at 240 °C with a sequence of 0.3/4.0/15.0/4.0 s for (TEMAHf) pulse/purge/oxygen plasma pulse/purge times, respectively. ALD of HfO_2_ was, thereafter, carried out using 120 cycles consisting of sequential Hf[N(C_2_H_5_)(CH_3_)]_4_-purge-O_2_ plasma–purge pulses.

In parallel, selected Si/TiN/SiO_2_/graphene stacks were coated with electron beam evaporated (EBE), thin SiO_2_ layers using a SiO_2_ pellet (99,8% nominal purity, BALZERS AG, Balzers, Liechtenstein). The process vacuum in the chamber was about 2 × 10^−6^ mbar at a rate of growth of 2 Å/s.

Annealing of selected ALD samples with SiO_2_ layers on Si/TiN/substrates was performed at 1000 °C in an Ar gas (99.999%, Linde Gas) environment for 10 min (after the temperature was ramped up from 500 to 1000 °C for 90 min) or at 600 °C in a vacuum (1.5 × 10^−5^ mbar) for 30 min.

### 2.4. Characterization

X-ray photoelectron spectroscopy (XPS) was exploited to evaluate the composition of SiO_2_ films before and after annealing (at 600 °C). The spectra were collected at normal emissions using a SPECSGROUP (Berlin, Germany) Phoibos150 hemispherical analyzer and a Prevac RS40B1 non-monochromatic dual anode X-ray source (Al-K_α_/Mg-K_α_). The minor sample charging was corrected by adjusting the adventitious carbon (typically present in ex situ-measured samples) C 1s peak to 285.2 eV. Spectral components were fitted, and elemental content from survey spectra was estimated using CasaXPS software [29].

The thicknesses of the deposited SiO_2_ and HfO_2_ films were evaluated by X-ray reflectometry (XRR) using Cu Kα radiation (SmartLaB, Rigaku, Tokyo, Japan), as well as by a spectroscopic ellipsometer using GES-5E (Semilab Co, Budapest, Hungary) with a micro-spot option where light is focused on a film surface via a telescope. The converging angle of the beam in the ellipsometer was about 4°, and the spot size was about 0.35 mm × 0.8 mm for a 65° angle of incidence. Fitting was performed using the program SEA (Semilab Co., Budapest, Hungary). Fit quality was characterized using a correlation function between the measured and computed spectra, R^2^.

To characterize the formation and integrity of graphene, a micro-Raman spectroscopic system Renishaw (Wotton-under-Edge, UK) inVia at an excitation wavelength of 514 nm was used. The spectral resolution reached 1.5–2 cm^−1^, and the Si reference was used for calibration. Raman spectra were measured after transferring graphene to SiO_2_ dielectrics and after deposition of the upper layer (SiO_2_ or HfO_2_) dielectrics on top of the graphene. All graphene band values were fitted by the Lorentz function.

The surface morphology of the graphene and dielectrics, as well as the cross-sectional investigation of the deposited films, were performed by high-resolution scanning electron microscopy (HR-SEM; FEI Helios NanoLab 600, Hillsboro, OR, USA). In order to perform cross-sectional studies, a thin lamella was prepared by a focused ion beam (FIB) in the same microscope. It was further studied in a scanning transmission electron microscopy (STEM) mode of SEM.

### 2.5. Electrical Measurements

For the electrical measurements, an about 2 µm-thick positive photoresist (AR-P 3510T, Allresist GmbH, Strausberg, Germany) was spin-coated (4000 rpm, 60 s) on the top of cleaned stack structures and then softly baked at 100 °C. Maskless photolithography (µMLA, Heidelberg Instruments Mikrotechnik GmbH, Germany), with the dose control of the light source at 390 nm, was used. After the exposition, the samples were chemically developed following a method analogous to that described earlier [30]. Thereafter, Ti electrodes were electron beam evaporated to thicknesses of around 100–110 nm at room temperature (RT) in a process vacuum of about 2 × 10^−6^ mbar at a growth rate of 2 Å/s. Finally, the lift-off procedure was carried out in a warm acetone bath. The Ti electrodes used in the measurement had diameters of either 50 or 250 µm. To provide ohmic contact, the backside of the silicon substrate was etched with a 7% hydrogen fluoride solution and covered with a thermally evaporated aluminum layer with a thickness of 100 nm at RT.

**Figure 1:**
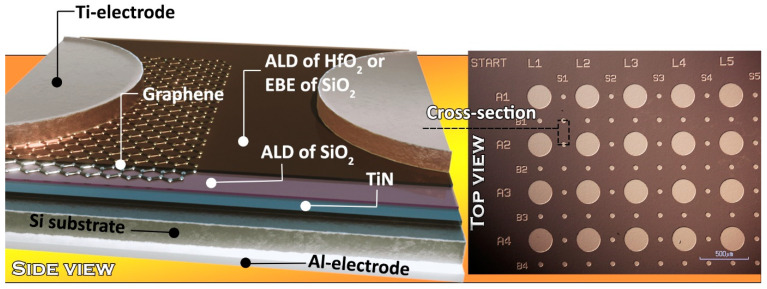
Graphic representation of the stacked nanostructures combined into one figure with and without graphene interface layers devised for resistive switching (**left panel**) and bird’s-eye-view through an optical microscope of the Ti electrode matrix fabricated on the topmost oxide layer by maskless photolithography (**right panel**).

The samples were electrically characterized at a probe station, at a Cascade Microtech (Beaverton, OR, USA) probe station MPS150, using a Keithley 2636A source meter (Keithley Instruments, Cleveland, OH, USA). The measurements were carried out in a light-proof and electrically shielded box. Figure 1 represents, graphically, the design of the stacks devised for the resistive switching (RS) measurements, as well as the microscopic image of the electrode matrix defining the size of the electrically measurable structures.

CVD-grown large-area graphene was inserted in order to separate two different dielectric layers. Graphene might act as a source of defects for the upper functional dielectric layer, which is necessary for resistive switching or, alternatively, graphene could act as an ion-blocking layer, which may provide opportunities to control ionic dynamics at the nanoscale, reported earlier [31].

Table 1 presents the sequence of the stacks prepared either with or without graphene interlayers. The rows in each of the table columns are summed up to denote nanostructures devised to evaluate the role of the graphene film as interface layers between the functional ALD-grown SiO_2_/HfO_2_ and ALD-grown SiO_2_/EBE-grown SiO_2_ dielectrics. The electrodes, i.e., TiN and Ti, allowing one to examine the appearance and stability of resistive switching, are also denoted.

## 3. Results and Discussion

### 3.1. Morphology and Growth

Some of the graphene films tended to break, i.e., delaminate when transferred to the ALD of the SiO_2_ dielectrics onto the Si/TiN substrates. A similar phenomenon has been observed before [4,32].

**Figure 2:**
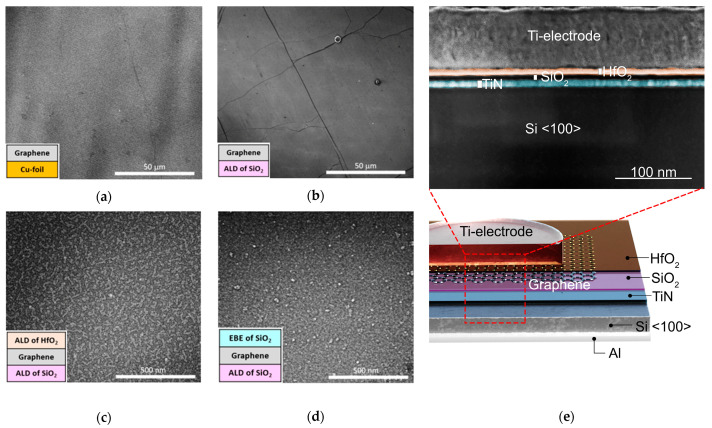
Bird’s-eye-view of SEM images of the surfaces of the graphene layer, as deposited on Cu foil (**a**), graphene transferred to ALD-grown SiO_2_ film (**b**), ALD-grown HfO_2_ film on graphene (**c**), electron beam evaporated SiO_2_ film on graphene (**d**), and cross-sectional image (the dark field, obtained in STEM mode of SEM) of a thin lamella prepared from the SiO_2_/Graphene/HfO_2_ (Sample 3 in Table 1) stack structure (**e**). Note that graphene is not visible in this image at this resolution. The oxide layers in the image have been false-colored to make the distinction between them “easier”.

SEM images of both the as-deposited (Figure 2a) and transferred graphene layers (Figure 2b) revealed an appreciable homogeneity of graphene on a microscopic scale. The SEM image of the as-deposited graphene (Figure 2a) shows uniform and continuous graphene coverage without visible discontinuities or defects. Thereby, the contrast between the features apparent in the image is mainly due to the steps on the underlying copper surface. After the transferring process, the graphene retained its homogeneous coverage of the substrate in spite of some folds, small ruptures, and residuals apparent on the surface (Figure 2b). Similar results have been demonstrated earlier in another study, where PMMA was used as a supporting layer [11].

The top HfO_2_ layer grown on the graphene exhibited a granular structure, which is indicative of a tendency for nucleation at spatially separated adsorption sites, probably structural defects, to be followed by the further growth of grains (Figure 2c). The surface of the EBE-grown SiO_2_ appeared rather featureless, except for some aggregated granular particles, as is characteristic of an amorphous film (Figure 2d).

Cross-sectional high-resolution SEM measurements revealed that the oxide layers constituting the stacked nanostructures are well distinguishable (Figure 2e). A similar cross-sectional distinction of the component layers has been demonstrated before; for instance, in the work of Savva et al. [33].

**Figure 3:**
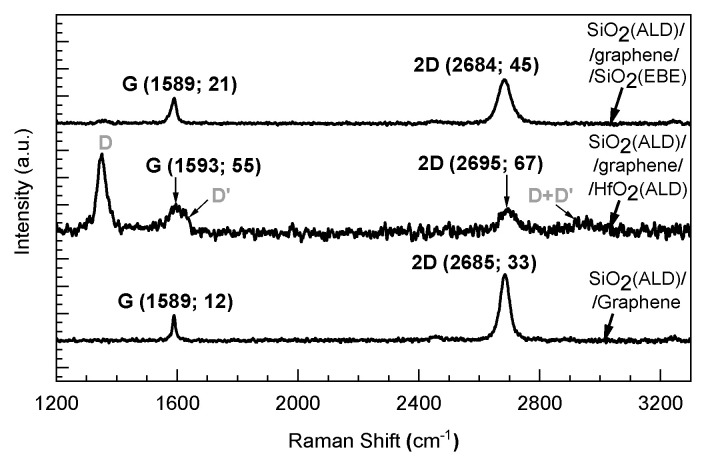
Typical Raman spectra of graphene-based stacked nanostructures. All spectra are normalized according to the G–band intensities. The positions and widths of the main bands (G and 2D) are indicated as labels in the spectra. The order of layers in stacked structures is also denoted by labels.

Due to the extremely small thickness of the graphene, micro-Raman measurements naturally occurred as the most adequate method, enabling the confirmation of the presence and quality of graphene in the stacked nanostructures built in the present study. The Raman spectrum of the graphene transferred on top of the ALD-grown SiO_2_ films on the Si/TiN substrates is depicted at the bottom of Figure 3. The average positions of the main G and 2D bands, defining the formation and presence of graphene, were 1589 cm^−1^ and 2685 cm^−1^, respectively. The band indicative of the presence of structural defects in graphene, i.e., the D-band, was not observable in the samples without a top HfO_2_ layer.

In separate preliminary experiments, attempts were made to grow SiO_2_ films on top of the transferred graphene layer using an earlier established process [27] where Si_2_(HNC_2_H_5_)_6_ and ozone were used as the cation and anion precursors, respectively. The precursor chemistry described in the latter study was also exploited in the present study to grow the base SiO_2_ film prior to transferring the graphene. However, the attempts to grow SiO_2_ films on top of the transferred graphene occurred unsuccessfully. The graphene was severely damaged in this process, as no G and 2D bands could be detected in the Raman spectra (not shown). Evidently, if the ALD temperature exceeds 40 °C, the ozone as the oxygen precursor is able to completely destroy the graphene in such a process. Similarly, the destructive effects of ozone on graphene have been observed in some earlier studies [22]. Nevertheless, in separate and complementary experiments, Si/TiN/SiO_2_/graphene nanostructures could be coated with evaporated physical vapor, i.e., EBE-grown SiO_2_ dielectrics, instead of ALD-grown SiO_2_. After the EBE of the SiO_2_ on the top of the graphene, only a low-intensity D-band emerged in the Raman spectra (Figure 3, the top spectrum). A rather wide band may be indicative of doping to some extent, being due to the dielectric-induced electron–hole charge fluctuations [34] or nanometer-scale strain variations in graphene [35,36]. In the case of HfO_2_ grown using plasma-assisted ALD with a protective buffer layer [4] on graphene, intense D and D′ bands, implying the formation of structural defects in the graphene, appeared in the present study, with an I_D_/I_D′_ intensity ratio varying from 7.0 to 8.0, together with an extra D + D′ band (Figure 3, the middle spectrum), which probably indicate vacancy-type defects [37]. Notably, despite the appearance of defects in the graphene, the integrity of the graphene layer was mainly retained after the plasma-assisted deposition of HfO_2_.

**Figure 4:**
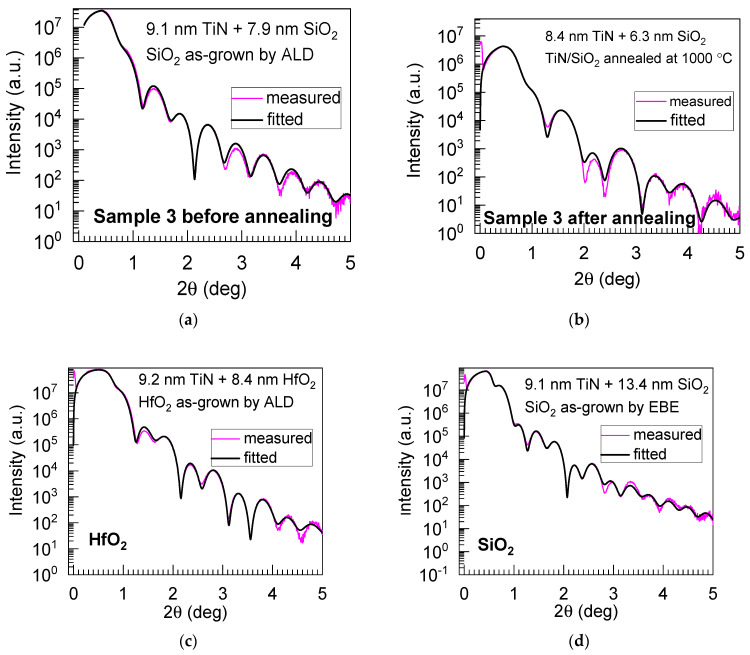
Measured and modeled X-ray reflectivity curves from SiO_2_ film, as grown by ALD (**a**), ALD-grown SiO_2_ annealed at 1000 °C (**b**), ALD-grown HfO_2_ (**c**), and EBE-grown SiO_2_ (**d**) on Si/TiN substrates. For the description of samples, see Table 1.

The thickness, density, and roughness of the dielectric films on the TiN, before and after annealing, were evaluated by X-ray reflectivity (XRR) measurements, with the results depicted in Figure 4.

Based on the XRR results, one can conclude that the SiO_2_ layer was noticeably densified upon annealing at 1000 °C, i.e., the layer thickness was decreased from 7.9 to 6.3 nm, and the density was increased from 1.76 to 2.26 g/cm^3^ (Table 1 and Table 2). After annealing at 600 °C, essential differences were not noticed, as the density change was about 2% (not shown). The comparable thickness values for the HfO_2_ (10.75 nm) and EBE-grown SiO_2_ (14.7 nm) films were obtained from ellipsometry measurements (Table 2), with a difference of ±2 nm, as compared to XRR.

**Figure 5:**
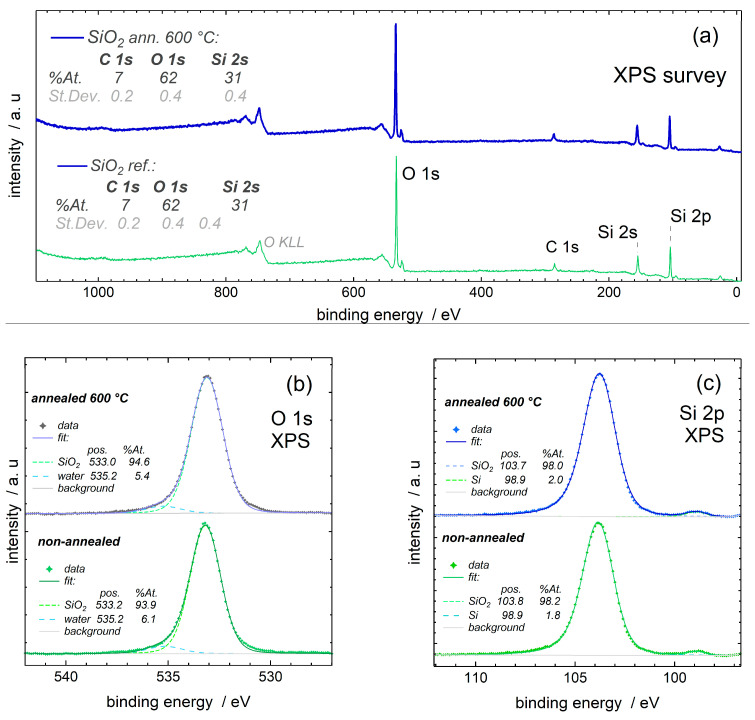
The survey (**a**) and elemental (**b**,**c**) XPS scans from the surfaces of non-annealed (as-deposited) and annealed SiO_2_ thin films. Positions of bands characteristic of oxygen (**b**) and silicon-containing compounds or elemental Si (**c**) are denoted by labels.

A selected SiO_2_ film in an as-deposited state, as well as a sample annealed at 600 °C, were subjected to XPS analysis in order to clarify whether the ozone-assisted ALD-produced SiO_2_ films could be described as silicon dioxide within a depth of a few nanometers from the film surface. The survey XPS spectrum (Figure 5) showed no stray or contaminant elements, which could be regarded as those originating from the growth process as residuals. An adventitious carbon signal was detected, though, as typical of ex situ samples at a level of ~7% at. along the expectedly thin, probing depth of XPS, and similarly for both the as-deposited and annealed samples.

The Si 2p (and 2s) photoelectron spectra are virtually identical, with a dominant oxide peak just below 104 eV of binding energy, which is in agreement with the literature’s values [38] but is also somewhat surprising, as they show a tiny elemental (non-oxidized) silicon feature (~2% of the overall Si 2p signal) at 98.9 eV of binding energy. The O 1s spectrum was centered slightly above 533.0 eV of binding energy, which fits well within the spread of the binding energy values reported earlier for SiO_2_ [38].

### 3.2. Resistive Switching Measurements

The devices subjected to resistive switching measurements were built on solid media consisting of SiO_2_/graphene/HfO_2_ (Samples 1, 3), SiO_2_/HfO_2_ (Samples 2, 4), and SiO_2_/graphene/SiO_2_ (Sample 5) layered stacks, as described in Table 1. 

**Figure 6:**
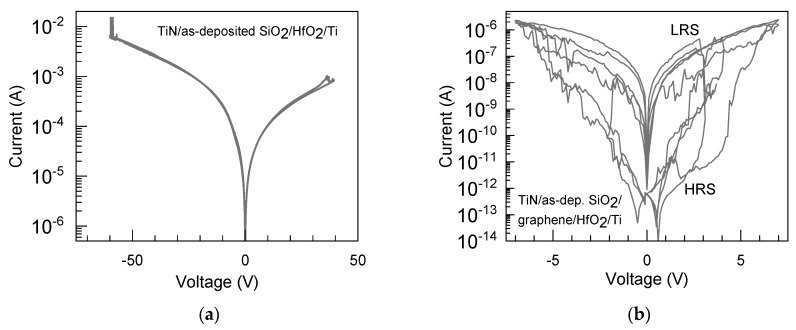
Current–voltage characteristics measured in resistive switching regime on TiN/SiO_2_/HfO_2_/Ti (Sample 2) (**a**) and TiN/SiO_2_/graphene/HfO_2_/Ti (Sample 1) (**b**) cell structures (Table 1) containing as-deposited SiO_2_ and HfO_2_ dielectric films. The diameter of the top titanium electrode was 250 μm. LRS and HRS denote the low- and high-resistance states, respectively.

The graphene-free TiN/SiO_2_/HfO_2_/Ti (Sample 2 in Table 1) devices could not switch resistively, plausibly due to the markedly high oxide quality and insulating properties of the solid medium between the electrodes. The applied voltage could be varied in both polarity directions between zero-voltage and as high as 40–50 V without evidence of reversible or irreversible breakdown events (Figure 6a).

At the same time, the performance of the TiN/SiO_2_/graphene/HfO_2_/Ti (Sample 1) stacks containing non-annealed SiO_2_ layers was markedly different compared to the devices without graphene. Figure 6b depicts the RS-like switching cycles recorded from the device containing stacks built on the as-deposited SiO_2_ (Sample 1). One can see that the currents still remained in the order of 1–10 pA, at voltages ranging from 0.5 to 1.5 V, towards both polarities in a high resistivity state (HRS). Further, the switching SET events could be recorded in the voltage range of 2.0–4.5 V, followed by the current–voltage envelope curves remotely characteristic of a switching device. The difference between low- and high-resistivity states (LRS and HRS, respectively) could extend even over 4 orders of magnitude (I_LRS_:I_HRS_ = 4600, on average). The switching mechanism was bipolar. However, the switching SET and also RESET events, as well as the LRS and HRS levels, occurred rather irregularly, suffering from abrupt changes in the switching direction (i.e., clockwise vs. counterclockwise). At the same time, the current–voltage curves implied possible multilevel RS performance. Such a “butterfly-like” appearance of the RS behavior is likely indicative of considerable structural and chemical inhomogeneities in the solid medium subjected to switching. It is possible that the irregular switching behavior is related to the defective structure of graphene, as revealed by the Raman spectrum from the same stacked medium (Figure 3, the middle spectrum). However, the intermediate graphene layer could serve as a source of defects, increasing conductivity and promoting switching. Despite irregularities, the switching behavior detected was indicative of the further possibility of optimizing the process with an appreciable low-to-high resistance ratio.

However, the quality of the upper oxide layers did not yet suffice for the provision of uniform switching in terms of both the voltages and state ratios.

**Figure 7:**
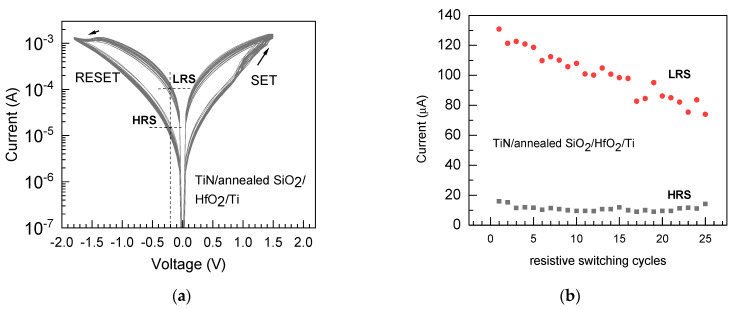
Current–voltage (**a**) and endurance (**b**) characteristics measured in a resistive switching regime on the TiN/SiO_2_/HfO_2_/Ti cell structure built on the TiN/SiO_2_ base stack annealed at 1000 °C (Sample 4 in Table 1). The low- and high-resistivity states, denoted by LRS and HRS, respectively, were recorded at reading voltages of 0.2 V.

Further measurements were performed on the TiN/SiO_2_/HfO_2_/Ti (Sample 4) cell structures built on the TiN/SiO_2_ base substrate annealed at 1000 °C but without intermediate graphene layers transferred onto the SiO_2_ film. The switching current–voltage curves, characteristic of an electrode-defined resistive switching memory cell, could repeatedly be recorded (Figure 7a). However, the endurance characteristic (Figure 7b) revealed that the stability of the low-to-high-resistivity ratios examined and recorded on different devices, i.e., measured from the top electrodes throughout the whole matrix, was rather poor, as the decrement in the LRS:HRS ratio (I_LRS_:I_HRS_, measured at 0.2 V) upon application of sequential switching voltage pulses had already approached an order of magnitude after few tens of the switching cycles.

**Figure 8:**
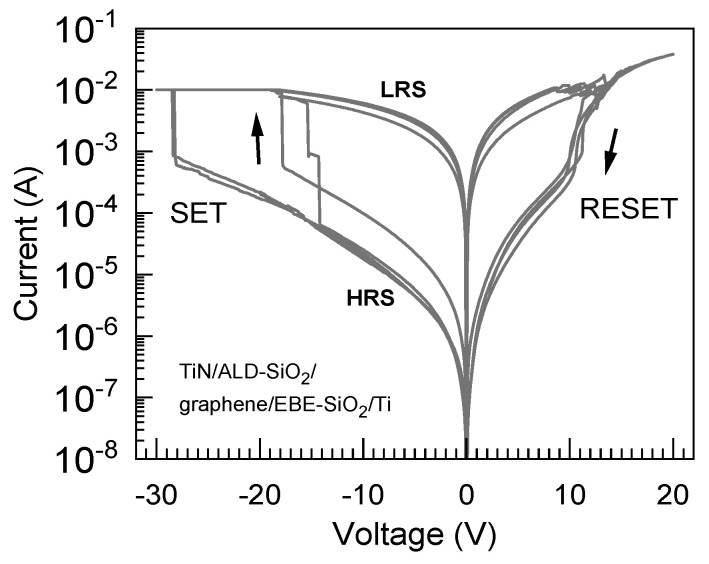
Resistively switching current–voltage curves measured from the TiN/SiO_2_/graphene/(EBE)SiO_2_/Ti devices (Sample 5 in Table 1).

Although the ALD process of SiO_2_ damaged the graphene layer, as mentioned above, the EBE of SiO_2_ on the graphene could still provide cells containing graphene between the SiO_2_ layers. Electrical measurements demonstrated that TiN/SiO_2_/graphene/(EBE)SiO_2_/Ti (Sample 5 in Table 1) stacks could also exhibit resistive switching (Figure 8). However, the SET events, i.e., the switching from the high-resistivity state to the low-resistivity state, took place at unexpectedly high voltages ranging from 15 to 30 V. Since the switching cycles were repeatable, together with the RESET events occurring between 10 and 15 V, the performance of the cell is to be regarded as one describing a resistive switching device despite the high switching voltages and asymmetry in the current–voltage characteristics.

Possibly, that kind of high-voltage behavior could be explained by the preservation of the structural integrity of graphene after the electron beam evaporation process of the SiO_2_ dielectric on top of the graphene, as seen in the Raman measurements above (Figure 3). This analogous phenomenon has been reported earlier by Lee et al. [31]. The latter study proposed that in the case of multilayer graphene, i.e., several single-layer graphene sheets transferred sequentially and stacked to avoid native defects (e.g., holes), the ion intermixing and ion transport should be completely blocked. Thereby, the devices could not be successfully formed, and resistive switching could not be achieved at bias voltages below 5 V [31].

**Figure 9:**
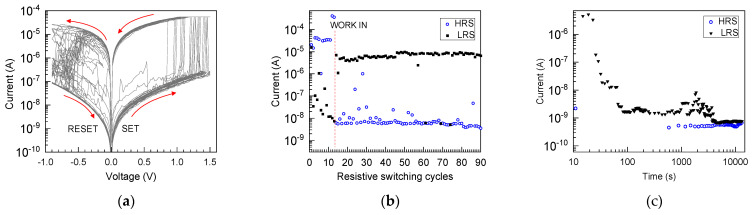
Current–voltage (**a**), endurance (**b**), and retention (**c**) characteristics measured in resistive switching regime on the TiN/SiO_2_/graphene/HfO_2_/Ti (Sample 3 in Table 1) cell structure built on the TiN/SiO_2_ base stack annealed at 1000 °C. The low- and high-resistivity states, denoted by LRS and HRS, respectively, were recorded at voltages of 0.2 V.

In the present study, further switching experiments were conducted after the ALD of HfO_2_ on top of the graphene layer. Notably, the switching performance of the TiN/SiO_2_/graphene/HfO_2_/Ti (Sample 3) devices (Figure 9) built on the annealed (at 1000 °C) SiO_2_ layer was improved in the sense of switching uniformity, compared to the devices containing an as-deposited SiO_2_ film. It is noteworthy that such devices did not necessarily even exhibit electroforming. This means that in some cases the work-in cycles providing the first SET and RESET events launching the further switching sequences were regarded as forming. As compared to the sample without intermediate graphene layers, the reliability and multiplicity of the current–voltage loops were increased (Figure 9a), and the tests also indicated a markedly higher endurance (Figure 9b) in terms of the number of switching cycles, as well as its state stability. At the same time, preliminary retention measurements (Figure 9c) indicated that reproducible, gradual self-resetting events could begin after 30 s, and the complete RESET occurred after an hour, along with the programming read pulse sequence. One can propose that the latter, if observed, could be described as volatile RS behavior.

It is also noteworthy that stacked structures based on SiO_2_ films grown to thicknesses exceeding 7 nm, both with and without graphene interface layers, could not perform as resistively switching devices. The annealing procedure had a useful effect only on stacked structures with thinner SiO_2_. These observations imply that the processing of switching cells requires further optimization and detailed studies, including those on the thicknesses of the constituent material layers and the annealing procedure. Further investigations should be aimed at the improvement of technically useful parameters, especially endurance and retention characteristics. Complementary, the resilience and mechanical durability of the graphene-containing layered stacks are to be studied in the future in order to evaluate the elasticity and adhesive properties of such stacks on electrode films.

## 4. Conclusions

It was shown that silicon oxide thin films grown by atomic layer deposition exploiting contemporary precursor chemistry, hexakis(ethylamino)disilane, and ozone could be used as base substrates for transferring graphene monolayers. X-ray spectroscopy on these silicon oxide films has revealed a negligible role for impurities and groups containing hydrogen or carbon in both as-deposited and annealed states. Graphene grown by chemical vapor deposition using methane and hydrogen can, upon a transfer process, be layered densely on silicon oxide as continuous films exhibiting only moderate, small-scale rupturing. Characteristic Raman bands as fingerprints of graphene have pointed to the appreciable integrity of the graphene film lacking bands, which are indicative of marked structural defect densities. Notably, the insulating dielectric material, hafnium oxide, can be grown using hafnium tetrakis(ethylmethyl)amide and oxygen plasma on top of the transferred graphene in the form of continuous layers to well-controlled thicknesses. The growth of homogeneous top oxide layers became favored after the pretreatment procedure of the graphene surface by a sequence of water pulses, followed by a sequence of metal precursor pulses at lowered temperatures. Remarkably, the plasma-assisted deposition did not destroy the integrity of graphene layers, although Raman bands referring to the creation of structural defects appeared in the spectra. At the same time, ozone-assisted deposition of silicon oxide on top of the graphene essentially damaged the graphene layer. In order to grow continuous silicon oxide on graphene non-destructively, electron beam evaporation had to be applied.

The stacks consisting of either silicon and hafnium oxide layers below and on top of the graphene or silicon oxide layers below and on top of the graphene demonstrated functionality in terms of their resistive switching behavior. The stacks mounted as switching media between the bottom titanium nitride and top titanium electrodes exhibited hysteretic current–voltage curves with a clear distinction between the high- and low-resistivity states. The endurance and retention tests allowed one to conclude that after additional optimization, the research can further confirm the utility of graphene in such devices. It is important to recall that the stacks of silicon and hafnium oxides, drawn up without an intermediate graphene layer, did not switch, plausibly due to their insulating capabilities and high breakdown fields.

## Figures and Tables

**Table 1:** Schematics of the nanostructured stacks expressed in separate columns, denoted by Samples 1–5. All stacks were mounted between ohmic Al electrode contacts on the bottom and Ti electrodes on the top. The rest of the constituent material’s layers, if present, are denoted by the corresponding chemical formulae. The temperature of the annealing procedure is denoted. The thicknesses of films, evaluated by X-ray reflectometry, are revealed by each material layer. The thickness measurement uncertainty is ±0.5 nm.

Steps	Sample 1 with Graphene	Sample 2 without Graphene	Sample 3 with Graphene	Sample 4 without Graphene	Sample 5
8	Ti top electrodes				
7	HfO_2_ (8.4 nm)	HfO_2_ (8.4 nm)	HfO_2_ (8.4 nm)	HfO_2_ (8.4 nm)	(EBE)SiO_2_ (13.4 nm)
6	Graphene	-	Graphene	-	Graphene
5	-	-	pre-annealed at 1000 °C		-
4	SiO_2_ (7.9 nm)	SiO_2_ (7.9 nm)	SiO_2_ (6.3 nm)	SiO_2_ (6.3 nm)	SiO_2_ (7.9 nm)
3	TiN (9.1 nm)	TiN (9.1 nm)	TiN (8.4 nm)	TiN (8.4 nm)	TiN (9.1 nm)
2	Si				
1	Al bottom electrode				

**Table 2:** Thickness, density, and refraction indexes of the studied dielectrics on Si/TiN substrates, estimated by XRR and ellipsometry methods. The ellipsometric thickness of (EBE)SiO_2_ was obtained using the predefined dispersion values, with the refractive index at 633 nm as 1.457.

Sample	XRR		Ellipsometry d (nm)
	d (nm)	ρ (g/cm^3^)	
SiO_2_ (Sample 1 and 2)	7.9	1.76	8.65
SiO_2_ + 1000 °C (Sample 3 and 4)	6.3	2.26	7.25
(EBE)SiO_2_	13.4	2.18	14.7
HfO_2_	8.4	9.33	10.75

## Data Availability

Not applicable.
